# The impact of within-host ecology on the fitness of a drug-resistant parasite

**DOI:** 10.1093/emph/eoy016

**Published:** 2018-06-27

**Authors:** Silvie Huijben, Brian H K Chan, William A Nelson, Andrew F Read

**Affiliations:** 1 Departments of Biology and Entomology, Center for Infectious Disease Dynamics, Pennsylvania State University, University Park, PA, USA; 2 Department of Biology, Queen’s University, Kingston, ON K7L3N6, Canada; 3 Department of Fogarty, National Institutes of Health, Fogarty International Center, Bethesda, MD, USA

**Keywords:** drug resistance, cost of resistance, within-host ecology, selection coefficient, epidemiological models, *Plasmodium chabaudi*

## Abstract

**Background and objectives:**

The rate of evolution of drug resistance depends on the fitness of resistant pathogens. The fitness of resistant pathogens is reduced by competition with sensitive pathogens in untreated hosts and so enhanced by competitive release in drug-treated hosts. We set out to estimate the magnitude of those effects on a variety of fitness measures, hypothesizing that competitive suppression and competitive release would have larger impacts when resistance was rarer to begin with.

**Methodology:**

We infected mice with varying densities of drug-resistant *Plasmodium chabaudi* malaria parasites in a fixed density of drug-sensitive parasites and followed infection dynamics using strain-specific quantitative PCR.

**Results:**

Competition with susceptible parasites reduced the absolute fitness of resistant parasites by 50–100%. Drug treatment increased the absolute fitness from 2- to >10 000-fold. The ecological context and choice of fitness measure was responsible for the wide variation in those estimates. Initial population growth rates poorly predicted parasite abundance and transmission probabilities.

**Conclusions and implications:**

(i) The sensitivity of estimates of pathogen fitness to ecological context and choice of fitness measure make it difficult to derive field-relevant estimates of the fitness costs and benefits of resistance from experimental settings. (ii) Competitive suppression can be a key force preventing resistance from emerging when it is rare, as it is when it first arises. (iii) Drug treatment profoundly affects the fitness of resistance. Resistance evolution could be slowed by developing drug use policies that consider in-host competition.

## Teaser

We found that drug treatment could double the fitness of a resistant parasite or increase it 10 000-fold, depending on in-host ecology and choice of fitness measure. Conversely, in the absence of treatment, the absolute fitness of resistant parasites was halved or completely eliminated when sensitive parasites were present.

## INTRODUCTION

A core aspiration of evolutionary medicine is to prolong the useful lifespan of antimicrobial drugs by slowing or even stopping the evolution of drug resistance [1]. When resistance first arises in a host, the rate of emergence of resistance in a patient and in a host population depends critically on the magnitude of the fitness costs and benefits of resistance. Fitness is a function of genotype, environment and genotype by environment interactions. Here, we concentrate on a key part of the environment: competition with sensitive co-infecting strains within a host. Competition between resistance and sensitive strains can occur when resistance first arises in a drug-sensitive infection, and in situations where hosts can become infected with several strains at the same time (co- or superinfections). Such co-infections are common in many disease systems, such as malaria (e.g. [2]), HIV (e.g. [3]) and bacteria (e.g. [4]).

In-host competition impacts the fitness of resistant pathogens in two ways. First, competitive suppression by drug-sensitive pathogens can prevent drug-resistant pathogens from rising to transmissible densities. Indeed, competitive suppression is likely the main reason why drug resistance is rare in the absence of drug treatment [5]. Second, drug treatment removes sensitive microbes. This allows populations of resistant pathogens to expand to fill newly vacated ecological space, a process called competitive release [6, 7]. Competitive release can be an extremely potent driver of resistance: imagine an infection in which resistant microbes make up <1% of the infection because of strong competition with sensitives. Removal of those sensitives by drug treatment could increase the abundance of resistant pathogens 100-fold. That expansion of the resistant parasite population due to the absence of competition is usually a far larger contributor of the rate at which resistance evolves in a population than the simple survival advantage conferred by resistance that plays out in hosts infected only with resistant parasites [8–13].

For some years, we have been using a mouse model of malaria to study competitive suppression and release of drug-resistant parasites [6, 14–18]. Here we report experimental estimates of the quantitative impact of competitive suppression and of drug-induced competitive release on the fitness of resistant parasites when there is variation in the clonal composition of the initial infection. The amount of resistance in an infection depends on a number of factors, such as how long a resistant strain has been in a sensitive population after a mutational event (or in the case of bacteria, by horizontal gene transfer), and the degree of co- and super-infection consisting of both resistant and sensitive strains within hosts. We hypothesize that competition with sensitive parasites causes a greater reduction in the fitness of resistant parasites when resistant parasites are initially rarer in an infection. This is because the smaller resistant populations are to begin with the smaller they will be when density-dependent regulation of the entire parasite population begins.

In pathogens, a variety of fitness surrogates are used to estimate fitness, including replication rates, pathogen titers (densities), persistence, infectious periods and transmissibility, as well as selection coefficients and relative growth rates during log growth phase. Each of these has it merits as a fitness measure, depending on the context and question. The aim of this study was to assess the magnitude of competitive suppression and release on these of fitness measures under different competitive scenarios using rodent malaria as a general model system. We estimated the quantitative impact of within-host ecology for all of these fitness measures and evaluated how the magnitude of fitness differences depended on the choice of fitness measure.

## METHODS

### Parasites and hosts

Two genetically distinct rodent malaria parasite clones of the species *Plasmodium chabaudi* were used in these experiments: drug-sensitive clone AJ_5p_ (hereafter referred to as clone S) and drug-resistant clone AS_6p(pyr-1 A)_ (hereafter referred to as clone R). Both clones were isolated from thicket rats and subsequently cloned [19]. Clone R was made resistant by a single high-dose exposure to pyrimethamine [20]. Hosts were 15-week-old female C57Bl/6 laboratory mice (Charles River Laboratories). All mice were kept on a 12:12 L:D cycle, fed Laboratory Rodent Diet 5001 (LabDiet, PMI Nutrition International) and received 0.05% para-aminobenzoic acid (PABA)-supplemented drinking water to enhance parasite growth [21]. 

### Experimental design, infections and drug treatment

The experiment consisted of either single infections of focal clone R, or mixed infections of clone R and clone S (Table 1). Only single infections of clone R were established to keep number of experimental mice down. The inoculum of clone S was kept constant at 10^6^ parasites per mouse in all treatment groups in which it was present. The inoculum of clone R consisted of 10^6^, 10^5^, 10^3^ or 10^1^ parasites. Note this experimental setup confounds infecting dose and the R:S clone ratio: infections initiated with the highest dose of clone R (1:1 R:S) began with roughly twice the number of parasites that were used to start the infections with the lowest dose of clone R (1:10^5^) R:S. Nevertheless, this design makes it possible to assess the magnitude of competitive suppression and release of focal clone R in the presence and absence of the other clone. We note that a 2-fold difference in total parasite dose has previously been shown to have a negligible effect on overall parasite dynamics [22]. Half of the mice were drug treated and the other half left untreated. Each treatment group consisted of five mice, except for the groups with an inoculum of 10^1^ resistant parasites that consisted of 10 mice to allow for the possibility that some mice failed to become infected because of stochastic loss due to the low inoculum size (Table 1).

**Table 1.** Experimental set-up

	Mixed infection			Single infection	
	R parasites	S parasites	n	R parasites	n
No drugs	10^6^	10^6^	5^a^	10^6^	5
	10^5^	10^6^	5^b^	10^5^	5
	10^3^	10^6^	5	10^3^	5
	10^1^	10^6^	10^a,b^	10^1^	10
Drugs	10^6^	10^6^	5	10^6^	5
	10^5^	10^6^	5^a^	10^5^	5
	10^3^	10^6^	5^a,b^	10^3^	5
	10^1^	10^6^	10	10^1^	10^a,a^

Groups were inoculated with clone R in the single infections, or clone R and clone S for the mixed infections. The inoculum of clone S was always 10^6^ parasites, the inoculum of clone R varied from 10^6^ to 10^1^ parasites. All treatment groups with an inoculum size of 10^1^ parasites had 10 mice at the start of the experiment. All other treatment groups consisted of 5 mice. Drug treatment was given on Days 6–9 post-infection.

a An excluded mouse.

b A dead or euthanized mouse.

Drug treatment started on Day 6 post-infection (PI), which is when pronounced anemia and weight loss begin to show [23, 24], and consisted of 8 mg/kg pyrimethamine dissolved in dimethyl sulfoxide (DMSO), administrated by intraperitoneal (i.p.) injection of 50 µl on four successive days. This drug has been first-line treatment for *P. falciparum* infections in combination with sulfadoxine but is no longer used clinically due to resistance, though still used as preventive treatment during pregnancy [22]. The dose in this study was previously demonstrated to clear *P. chabaudi* infections [6]. The untreated mice received i.p. injection of DMSO-only contemporaneously.

### Monitoring of infections

Asexual parasite density (in-host replicative stages) and gametocyte density (transmissible stages) of both clones were measured daily (Days 3–21 PI) and three times a week thereafter (Days 23–49 PI). For each mouse at any time point, 2 µl of blood was taken by tail snip for red blood cell density measurements using flow-cytometry (Beckman Coulter). Another 5 µl of blood was taken for DNA extraction, which was used to estimate total parasite density (asexual parasites and gametocytes) by quantitative PCR using clone-specific assays. A further 10 µl of blood was taken and lysed immediately for RNA extraction. Quantitative PCR was performed on cDNA, based on expression of a common gametocyte gene, using the same clone-specific assays used to estimate gametocyte density from extracted DNA. Asexual parasite density was estimated by subtracting the gametocyte density from the total parasite density [23]. PCR protocols are given elsewhere [17], using the PCR conditions as described by Bell *et al.* [24].

### Fitness measures

Various proxies of fitness were calculated. Our main focus was on the absolute fitness, as opposed to relative fitness, of the resistant parasite because that is what determines the rate of evolution of resistance in patients and in host populations when resistance is rare [7]. The proxies of absolute fitness we used are defined in Table 2. *Plasmodium chabaudi* parasites invade red blood cells, replicate and then synchronously rupture once a day; hence our focus on summing or averaging across days, and on daily rates of increase.

**Table 2.** Various proxies of absolute fitness calculated for each infection for sensitive, resistant or all parasites and throughout the infection, during treatment or after treatment, as stated in the main text

Variable	Quantitative definition	Unit
Initial growth rate	Slope of regression of parasite density by time for Days 3–6 post-infection	Daily fold-increase
Parasite density	Sum of asexual parasite densities, either throughout the infection (Days 0–49) or post-treatment (Days 10–49)	10^6^ parasites/µl blood (whole infection) or 10^4^ parasites/µl blood (post-treatment)
Parasite persistence	Day post-infection of last detectable asexual parasites by PCR from either start of the infection or after treatment ended	Days
Infectious period	Number of days of detection of gametocytes by PCR from either first to last day of detection or from at minimum end of treatment to last detection of gametocytes	Days
Infectiousness	Mean daily probability of onwards transmission to mosquitoes each day, predicted from gametocyte density-infectivity function described by [65] calculated either throughout the infection (Days 0–49) or post-treatment (Days 10–49)	Probability

To better understand the fitness difference among strains when they are competing, it is common to estimate relative fitness (fitness R/fitness S) using strain-specific growth rate [25, 26]. We estimated relative fitness using the coefficient of selection on the resistant clone during infections. The selection coefficient is the difference in the *per capita* growth rate of the resistant clone and the susceptible clone. Growth rates for each clone vary through the course of mixed infections and so we calculated the instantaneous selection coefficient from the frequency of the competitors as described by Huijben *et al.* [17]. Selection coefficients were calculated only for asexual parasite densities since too few data points with positive values for gametocytes of both clones at any one time were available for reliable model fitting.

### Statistical analysis

General linear modeling on fitness estimates from Table 2 was performed in R 3.2.0 [27] with the following factors: ‘competition’ (clone S present or absent), ‘drugs’ (treated or untreated) and ‘R-inoculum’ (10^6^, 10^5^, 10^3^, 10^1^). Maximal models were fitted first and, beginning with higher order interaction, non-significant terms were sequentially removed to generate minimal models. Significance versus non-significance was based on a Type I error rate of 5%.

For the following reasons, several mice had to be excluded from data analysis (Table 1) but the raw data from all mice are shown in the Supplementary data. Three mice died or were euthanized during the infection (in the groups untreated mixed 10^5^ R-inoculum, untreated mixed 10^3^ R-inoculum, drug treated mixed 10^3^ R-inoculum) and were removed from further analysis. Another six mice were additionally excluded from the analysis. Three of these failed to become infected with resistant parasites; two from the low single inoculum of 10^1^ R parasites (both drug treated single 10^1^ R-inoculum) and one in the untreated mixed 10^1^ R-inoculum group. The lack of resistant parasites in this latter mouse could have been a competition effect, but being conservative, it was excluded from analysis. One further mouse (drug treated mixed 10^3^ R-inoculum) failed for unknown reasons to respond to drug pressure and two mice received an inoculum of several orders of magnitude lower than intended, as judged by the pre-treatment infection kinetics (untreated mixed 10^6^ R-inoculum, drug treated mixed 10^5^ R-inoculum, Table 1). Most likely because of sampling error at very low density, estimates for the initial growth rate of resistant parasites in four of mice yielded biologically unreasonable values (10- to 25-fold per day, when it has to be less than 8-fold because each parasite in an infected red blood cell releases up to 8 progeny parasites [28]. These four estimates were excluded from the analysis of initial growth rates.

Note that a subset of the data from this experiment has been published elsewhere [64]. The aim of that earlier analysis was to determine the impact of the frequency of resistance in an infection on the probability of treatment failure, and so it concentrated only on the mixed infections. Here we focus on the fitness of the resistant parasites in mixed and single infections, to estimate the impact of competitive suppression and competitive release on the fitness of the resistant clone.

## RESULTS

Drug treatment and inoculating dose had no impact on the density of resistant parasites when grown alone (Supplementary Fig. S1). Thus, the resistant parasites were fully resistant to the drug dosages we used and any effect of treatments in the presence of multi-clonal infections is the consequence of competition with the susceptible strain.

### Competitive suppression

**Figure 1.**
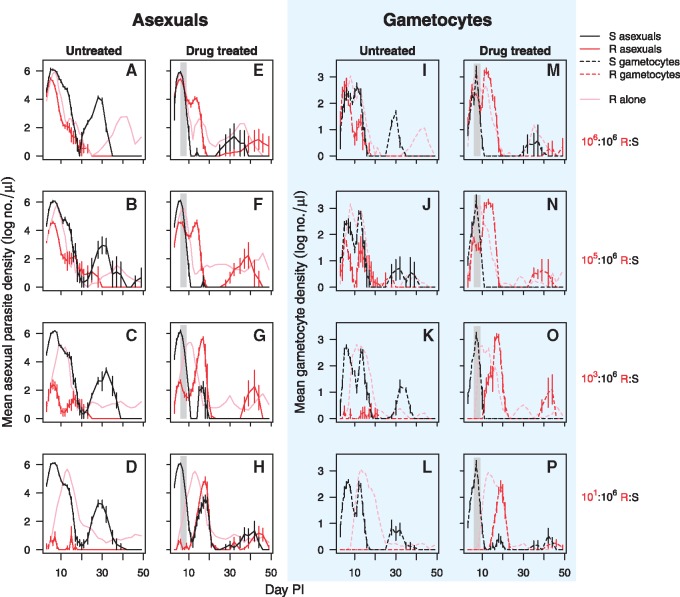
Within-host dynamics of mixed clone infections. Asexual parasite densities (solid lines—left two columns) and gametocyte densities (dashed lines—right two columns in blue shaded area) of drug sensitive clone S (black lines) and drug resistant clone R (red lines) in mixed infections that were untreated (first and thirds columns) or drug-treated (second and fourth columns). Drug treatment was given on Days 6–9 post-infection as indicated by the shaded area. Infections were inoculated with a clone R:S relative abundance of 10^6^:10^6^ (top row), 10^5^:10^6^ (second row), 10^3^:10^6^ (third row) and 10^1^:10^6^ (bottom row). In light pink, the asexual parasite and gametocyte mean dynamics of clone R when in infections without sensitive parasites (full data in Fig. S1). Data are geometric means (±SEM) of up to five mice for R:S abundance of 10^6^:10^6^, 10^5^:10^6^ and 10^3^:10^6^, or up to 10 mice for R:S ratio 10^1^:10^6^ (Table 1)

**Figure 2.**
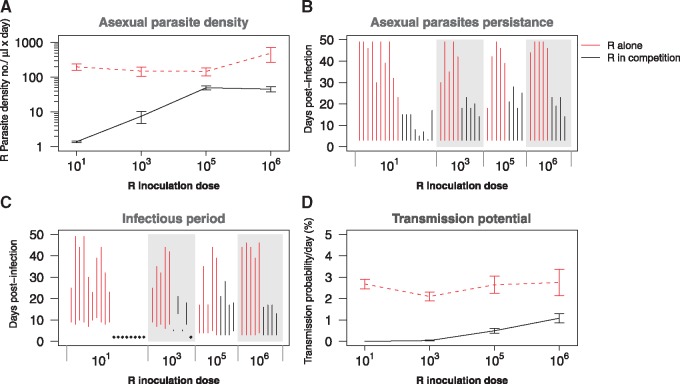
Competitive suppression—comparative performance of clone R alone (red lines) and in competition (black lines) in the absence of drug treatment. (**A**) Geometric mean asexual parasite density of clone R at different initial parasite dosages. (**B**) Duration of infection of resistant parasites (time to last detection by qPCR) for the different R inoculum sizes. Each line represents the duration in a single mouse. (**C**) Duration of infectious period (from first to last detection of gametocytes by qPCR) for the different R inoculum sizes. Diamonds represent infections that did not produce any gametocytes; again, data for each mouse are shown. (**D**) Mean transmission probability per day at different initial parasite dosages based on a gametocyte density-infectivity function [65]. Data in (A) and (D) are means (±SEM) from the entire infection period (Days 3–49 PI) of up to five mice (R inoculum sizes 10^6^, 10^5^, 10^3^) or up to 10 mice (inoculum size 10^1^; Table 1)

In untreated infections, the presence of susceptible parasites substantially reduced the fitness of resistant parasites (Fig. 1; Supplementary Fig. S2 and Table S1a). Competition reduced resistant parasite densities by 75–99.9% and infection durations by 40–75% (Fig. 2A and B; Supplementary Table S1a). Competition also shortened average infectious periods by 34–100%, and more than halved or eliminated altogether the probability resistant parasites would be transmitted (Fig. 2C and D; Supplementary Table S1a). The impacts of competition were especially pronounced in infections in which resistance was initially rare (Supplementary Table S1a), even though inoculation density had only a modest impact when resistant parasites were grown alone (3-fold or less, Supplementary Table S1a).

### Competitive release

At the start of treatment (Day 6 PI), the initial inocula of 10^6^, 10^5^, 10^3^, 10^1^ of clone R in mixed infections had become, respectively, 10^5.4^, 10^4.6^, 10^2.6^ and 10^0.^^7^/µl blood, corresponding to 34, 2.8, 0.028 and 0.0004% of the total parasite population.

**Figure 3.**
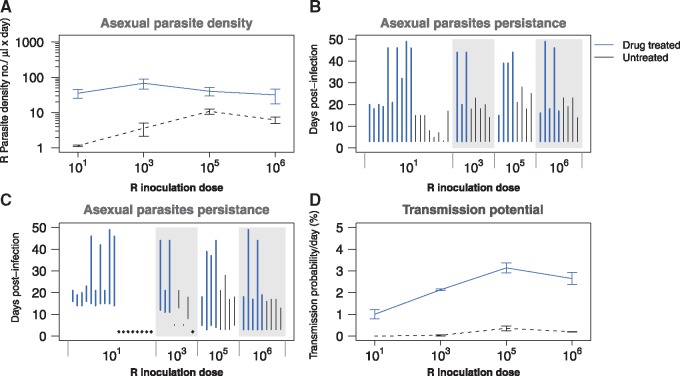
Competitive release—comparative performance of clone R in competition in the presence (blue lines) and absence (black lines) of drug treatment. (**A**) Geometric mean asexual parasite density of clone R at different initial parasite dosages. (**B**) Duration of infection of resistant parasites (time to last detection by qPCR) for the different R inoculum sizes. Each line represents the duration of one single infection. (**C**) Duration of infectious period (from first to last detection of gametocytes by qPCR) for the different R inoculum sizes. Diamonds represent infections that did not produce any gametocytes. (**D**) Mean transmission probability per day at different initial parasite dosages based on a gametocyte density-infectivity function [65]. Data in (A) and (D) are means (±SEM) from the post-treatment infection period (Days 10–49 PI) of up to five mice (R inoculum sizes 10^6^, 10^5^, 10^3^) or up to 10 mice (inoculum size 10^1^; Table 1)

Drug treatment rapidly cleared susceptible parasites (Fig. 1). As a result, the initially suppressed populations of resistant parasites were able to expand (Figs 1 and 3A; Supplementary Fig. S2), causing a pronounced second wave of parasites (Fig. 1—drug treated column). Drug treatment increased resistant parasite densities 30- to 500-fold (Fig. 3A), and resistant populations were able to persist 2–14 times longer after drug treatment (Fig. 3B; Supplementary Table S1b). Drug treatment greatly increased the densities of gametocytes produced by the resistant clone (Fig. 1 right hand panels) and more than doubled its infectious period (Fig. 3C; Supplementary Table S1b). Consequently, the probability that resistant parasites could transmit increased by one and often several orders of magnitude (Fig. 3D; Supplementary Table S1b). The population expansions and increased transmission probabilities were particularly pronounced for those treatments in which the resistant population was rare at the time of treatment (Supplementary Table S1b).

The preceding analyses focus on the post-treatment fitness of resistant parasites. The ecological details leading up to treatment will impact total fitness, which is an aggregate of performance before, during and after treatment. For example, when the resistant clone was more abundant at the time of treatment it was already producing a substantial number of gametocytes. Moreover, it was also at higher parasite densities, enabling it to capitalize more readily on the competitive release (Fig. 1, second and fourth column). For completeness, we therefore re-calculated our fitness measures for the entire duration of the treated infections. The net effect of this was that while drug treatment always greatly increased the transmission potential of the resistant parasites, the resistant parasites had the highest transmission potential where resistant parasites were most abundant when treatment began (Fig. 3D; Supplementary Table S1c). In fact, this maximum was as large as achieved in the complete absence of competition (Fig. 1, cf. Supplementary Table S1a and c). In contrast, the overall transmission potential of resistant parasites from low dose inocula remained less than they achieved alone, despite having the larger proportionate benefits of competitive release (cf. Supplementary Table S1a and c).

### Relative fitness

**Figure 4.**
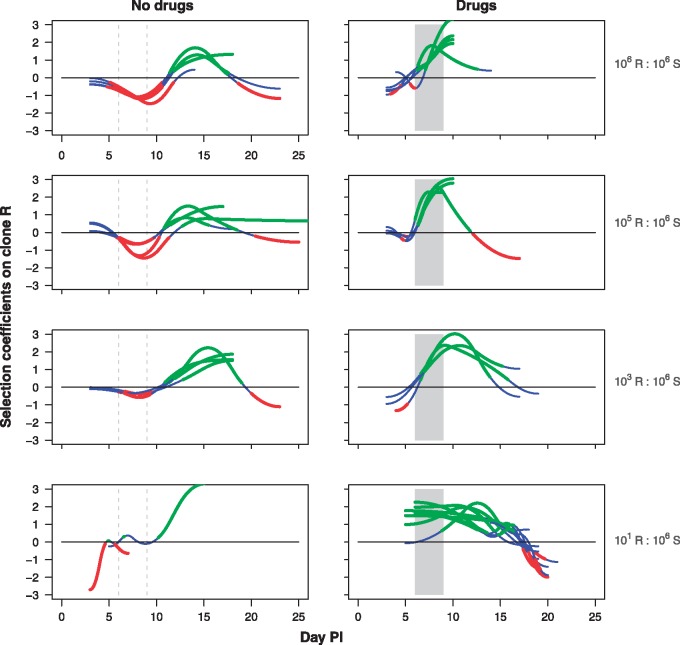
Selection coefficients over time. Selection dynamics on asexual resistant parasites for each mouse in mixed infections that were inoculated with a clone R:S relative abundance of 10^6^:10^6^ (top row), 10^5^:10^6^ (second row), 10^3^:10^6^ (third row) and 10^1^:10^6^ (bottom row). Infections were either untreated (left panels) or drug treated (right panels). Drug treatment was given on Days 6–9 post-infection as indicated by the shaded area. Mean selection dynamics are shown with blue segments denoting times when selection is not statistically different from zero, red segments times when selection is statistically less than zero, and green segments times when selection is greater than zero. Selection could be calculated up to the last day that both clones were detectable, which varied between mice. In 8 out of the 10 mice in the untreated 10^1^ R:10^6^ S group, too few data points with both clones above detection level were available to calculate the selection coefficients

Selection coefficients, which measure the instantaneous rate of change of resistant parasite density relative to the sensitive population, varied through the course of the infections (Fig. 4, left panels), with resistance in untreated mice under negative or neutral selection for the first 10 days, particular where resistance was initially common. That negative selection was followed by a period of positive selection for the next 7–10 days due to rapid declines of the sensitive parasites. In drug-treated mice, selection coefficients for resistant parasites were strongly positive once drug treatment began, and in many mice went to infinity as susceptible parasites were cleared (Fig. 4, right panels). In infections in which resistant parasites were rare when treatment started, susceptible parasites were able to recover when treatment stopped (Fig. 1G and H), reducing the selection coefficients on resistance (Fig. 4). Nevertheless, resistant parasites remained under positive selection for a week or more after drug treatment stopped.

### Relationship between initial growth rates and fitness

**Figure 5.**
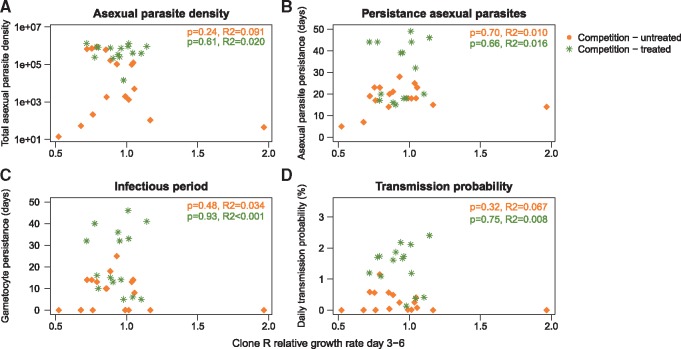
Relationship between initial growth rates and fitness. Association between initial relative growth rate of clone R (in competition) compared to growth rate of clone S (days 3–6 post-infection) with four clone R fitness parameters: A) Total asexual parasite density, B) asexual parasite persistence, C) infectious period and D) transmission probability. Each symbol represents a single mouse, orange diamonds are untreated infections, green asterisks are drug treated (days 6–9 post-infection) infections. Level of significance from linear regression of the slope and associated R-squared values are given for both untreated (top line) and drug treated (bottom line) infections

Relative growth rate of resistant parasites compared with susceptible parasites early in the infection were poorly predictive of the absolute fitness of the resistant parasite (Fig. 5), including the total number of resistant parasites as well as the infectious period and transmission probabilities, the key determinant of the rate of spread of resistance. Even the absolute rate of initial growth of the resistant clone poorly predicted the subsequent fitness of the resistant clone (Supplementary Fig. S6). This is because parasite persistence, infectious periods and transmissibility are more closely linked to parasite replication after the initial exponential growth phase (Supplementary Fig. S7).

## DISCUSSION

The rate of resistance emergence within individual hosts, as well as the spread of resistance in host populations, is arguably determined mostly by the absolute fitness of resistant microbes [7]. We found that the absolute fitness of our resistant parasite clone was profoundly affected by the presence of sensitive parasites, especially when resistance was rare to begin with. Depending on initial conditions, competition with sensitive parasites reduced the densities of resistant parasites by up to >99.9% and reduced the probability that resistance would be transmitted by 60–100% (Fig. 2; Supplementary Table S1). Drug treatment removed this suppression, leading to increased density of resistant parasites by several orders of magnitude and enhanced probability that resistance would be transmitted by 50-fold or more. The most marked relative increases occurred when resistance was rare at the time of treatment (Fig. 3). Thus, the absolute fitness of resistant parasites in the presence and absence of drug treatment, the key driver of resistance evolution, is highly dependent on the within-host ecological context. Ecological context-dependent fitness costs to being resistant have been reported in other systems, including phage resistance in a pathogenic bacterium of plants [29] and in drug-resistant bacteria of humans [25, 30].

An important determinant of drug resistance evolution is the fate of *de novo* resistant mutations. Our data suggest that competition with sensitive progenitors will play an important role in determining whether a *de novo* resistant mutant can be transmitted from hosts within which they occurred (compare Supplementary Fig. S1 and Fig. 1L and P). Resistant parasites starting at a 10^1^ inoculum in mixed infections were at a frequency of ∼10^−5^ at the time of drug treatment. There they did not produce a single detectable transmission stage in the absence of treatment. For a true *de novo* mutational event, the frequency of the initial mutant is likely closer to 10^−9^ [31]. Given that competitive suppression increased with rarity, and that *de novo* mutants must share essentially the same niche as the progenitor population, it seems likely that competitive forces acting against *de novo* mutants will be many-fold more intense than reported here. Quite possibly, competitive suppression is the major force preventing the emergence of any resistance that occurs *de novo*.

### Methodological implications

In a wide variety of experimental systems, a popular measure of pathogen fitness is some measure of replication rate during exponential (density-independent) growth phase, often measured *in vitro* (reviewed by [25, 26]; examples [30, 32–34]). We recognize that ‘mice are not men’ (as discussed, for example, in [16]) and rodent malarial parasites are not representative of all pathogens. Nevertheless, we suggest our data caution against strong inferences from surrogate fitness measures made where pathogens are not competing in a density-dependent manner. In our system at least, key fitness components such as parasite population size, persistence and transmission are poorly predicted by performance during exponential growth (Fig. 5, Supplementary Figs S6 and S7). Relating fitness components to fitness itself is challenging in any setting [35–39], but in the infection context, it is quite possible that rate of cell division in density-independent conditions fails to capture quantitatively more important determinants of pathogen fitness. Where strong inference is being made from experimental measures of fitness surrogates, there is a case for ensuring that these really are meaningful measures of fitness, as has been done in other contexts [40].

The importance of ecological context and choice of fitness measure is particularly acute when experimentally estimating the fitness costs of resistance. Costs of resistance are a main determinant of selection on resistance in the absence of drug treatment [25], and numerical estimates of cost are therefore a key parameter in models of drug resistance evolution. In the malaria context, for example, estimates of the cost of resistance range from 0 to 60% (e.g. [41–43]). Our two parasite clones were not isogenic, and so it is difficult to attribute any differences between them to resistance or other genetic traits. Nevertheless, we offer the following observations. Grown by themselves, the resistant and susceptible clones used here did not have statistically different growth rates, peak parasite densities or overall parasite densities (Supplementary Fig. S3), consistent with a cost of resistance of 0%. However, in those same mice, resistant parasites produced 31% fewer transmission stages than sensitive parasites (Supplementary Fig. S3B), consistent with a cost of resistance of 31%. In mixed infections, the resistant clone grows ∼10% slower than the resistant clone during the initial phase (Supplementary Fig. S5 and Table S1e), consistent with a cost of resistance of ∼10%. But that relative fitness differences of 10% is dwarfed by the 50–100% fitness differences introduced by competition and numerical abundance (Figs 2 and 3; Supplementary Table S1) consistent with costs of resistance of up to 100%.

Thus, depending on the competitive setting and fitness measures used, our data are consistent with strain differences ranging from 0 to 100%. Most of this variation is caused by density-dependence early in infections comprised of two strains. We contend that it is critically important to estimate costs in density-dependent settings: (i) competition can substantially amplify small differences in performance, as we demonstrated here, and (ii) costs of resistance can play out in terms of density-independent replication rates and in terms of competitive ability (e.g. [39, 44]). As such, comprehensive measures of resistance costs need to incorporate both competitive ability and density-independent growth. We are not the first to show that competition amplifies the costs of resistance (e.g. [45]), but our data clearly show that the magnitude of that amplification depends on the ecological details within hosts. This is not simply a question of *in vivo* versus *in vitro* measures. Indeed, for some fitness measures, there can be some agreement between estimates of the cost of antibiotic resistance from laboratory experiments and murine infection models [26]. Our point is that both density-independent and density-dependent processes play out during an infection, and assays capturing only a subset of those processes can be expected to give reliable quantitative estimates of the fitness costs and benefits of resistance. This logic suggests it is impossible to capture field-relevant competition experimentally, at least in human infections. It may be that the only meaningful estimates of costs of resistance are those observed in the clinical or epidemiological context in which they play out as opposed to laboratory settings (e.g. HIV [46] and TB [47]). Such measures also take account of the wide variety of other factors, such as genetic background [48], compensatory mutations [49], multiplicity of infection [10], drug dose [16, 17] and timing of drug treatment [6, 16].

### Implications for medicine and public health

Our data show that depending on the within-host ecology, drug treatment can cause resistant populations to expand up to 500-fold within a host and increase the likelihood of resistance transmission by at least 100-fold. It seems highly likely that competition occurs in a wide variety of pathogens [50, 51] but the magnitude of the fitness gains experienced by drug-resistant pathogens remains to be determined in other settings. Indeed, there will be pathogen–drug combinations where resistance does not confer a cost or is even advantageous in the absence of the drug (e.g. [39, 52]). Understanding how different treatment regimens impact both patient health and competitive release should help identify regimens which successfully treat patients without maximizing the emergence and onward spread of resistance [5, 7, 18, 53]. For example, over-treatment, when more drug is used than is required clinically, may have undesirable properties. In our experiments, the effect of treatment on increasing the resistant strain persisted long after the treatment had stopped (Fig. 5; pyrimethamine has a half life of hours in mice). We assume that this is because treatment made sensitive parasites rare, so that they become competitively suppressed by the numerically dominant resistant population. More intermittant dosing schedules might prevent that competitive reversal while still containing the total population [44]. It may even be possible to manipulate competition during treatment to slow or even prevent the emergence of resistance following treatment [54]. 

Our experiments exploit a rodent malaria model, yet our conclusion that in-host competition with sensitives is a key determinant of fitness of resistant is very likely generalizable across many pathogens. For the specific case of malaria, there is strong correlational evidence that competitive suppression and release occurs in human infections [2, 55]. Competitive suppression may explain why global establishment of high level resistance in *P. falciparum* malaria parasites against both chloroquine and sulphadoxine–pyrimethamine was due to vast selective sweeps from only a handful of independent *de novo* origins (reviewed in [56]). Certainly, multiple independent mutations are required for a viable parasite, and the failure of newly arisen mutants to spread because of stochastic loss is frequently discussed [10, 57–59]. Our data suggest that *de novo* mutations will most often be competitively excluded, and so to have any chance of reaching transmissible densities in a person, they would have to occur during or immediately before treatment.

The ultimate objective of many malaria policy makers and funding bodies is malaria elimination or even eradication [60, 61]. To a large extent, this ambition is dependent on effective drugs. Yet, we know surprisingly little about how the rate of spread of resistance is impacted by extensive drug administration, particularly mass drug administration (in which everyone in a population is simultaneously treated regardless of their infection status). Our data point to the critical importance of competitive release when resistance is rare in an infection. Yet, this is hard to study directly. Highly sensitive detection techniques are needed to provide important information on competitive interactions between resistant and sensitive *P. falciparum* strains in field situations [62, 63]. Standard PCR assays used on field samples would struggle to detect resistant parasites at time of treatment in all but the 10^5^ and 10^6^ treatment groups in the current experiment [64]. Nonetheless, a more thorough understanding of the determinants of in-host competition would help evaluate the evolutionary risks associated with treating patients who are not sick.

## Supplementary data

Supplementary data is available at *EMPH* online.

## Supplementary Material

Supplementary Data
