# Supplementary material for: The impact of within-host ecology on the fitness of a drug-resistant parasite
Source: Evol Med Public Health. 2018 Jun 27;2018(1):127–37. doi: 10.1093/emph/eoy016 (PMC6061792; doi:10.1093/emph/eoy016)
Supplement: Supplementary Data [file eoy016_supp.pdf]

Supplementary Information

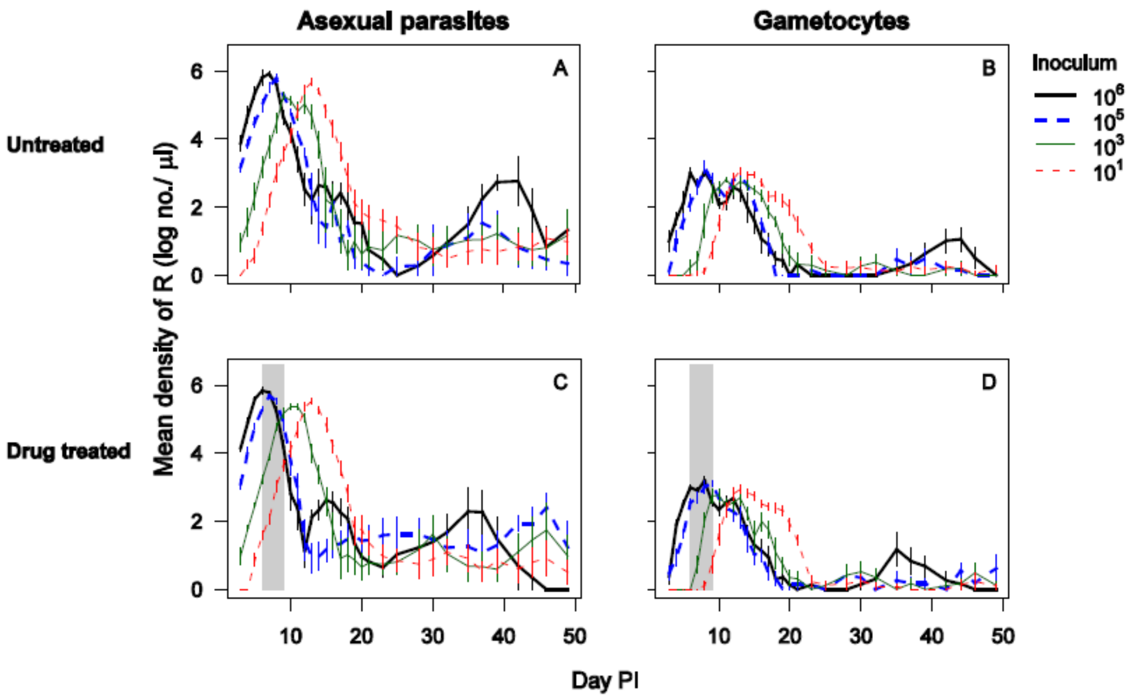

Figure S1. Asexual parasite densities (left panels) and gametocyte densities (right panels) in untreated (top panels) and drug treated (bottom panels) single infections of drug resistant clone R. The infections were initiated with an inoculum of 10^6 (thick solid black line), 10^5 (thick dashed blue line), 10^3 (thin solid green line) and 10^1 (thin dashed red line) parasites. Drug treatment was given on days 6-9 post-infection as indicated by the grey rectangle. Data are means (± standard error) of 5 mice (R-inoculum 10^6, 10^5, 10^3) or 10 mice (R-inoculum 10^1). Lower doses of clone R resulted in a delay in parasitaemia and gametocytaemia of about a day for each order of magnitude, but had no effect on asexual parasite and gametocyte densities ($F_{3,43}$=2.5, p=0.07, $F_{3,43}$=1.3, p=0.29 respectively). Drug treatment did not affect resistant asexual or gametocyte densities ($F_{1,43}$=0.1, p=0.75; $F_{1,43}$=1.4, p=0.25 respectively).

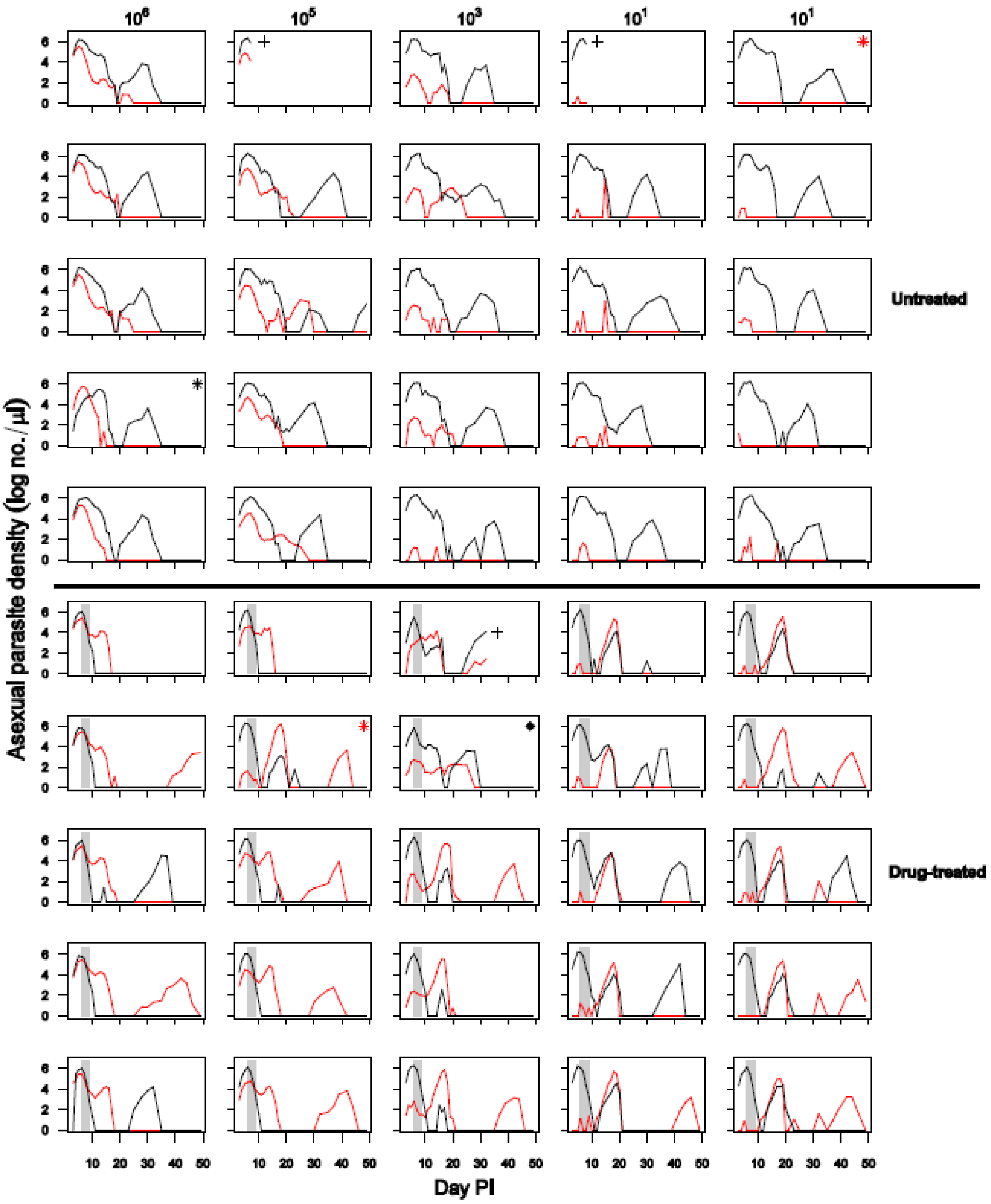

Figure S2. Asexual parasite densities of drug-sensitive clone S (black lines) and drug-resistant clone R (red lines) in mixed infections that were untreated (upper half) and drug treated (lower half). Drug treatment was given on days 6-9 post-infection as indicated by the shaded area. Infections were inoculated with 10^6 parasites of clone S and 10^6 (left column), 10^5 (second column), 10^3 (middle column) or 10^1 (last two columns) parasites of clone R. Asterisks denote mice excluded from the analysis (see methods), crosses denote mice that died or were euthanized during the experiment and were also excluded from the analysis.

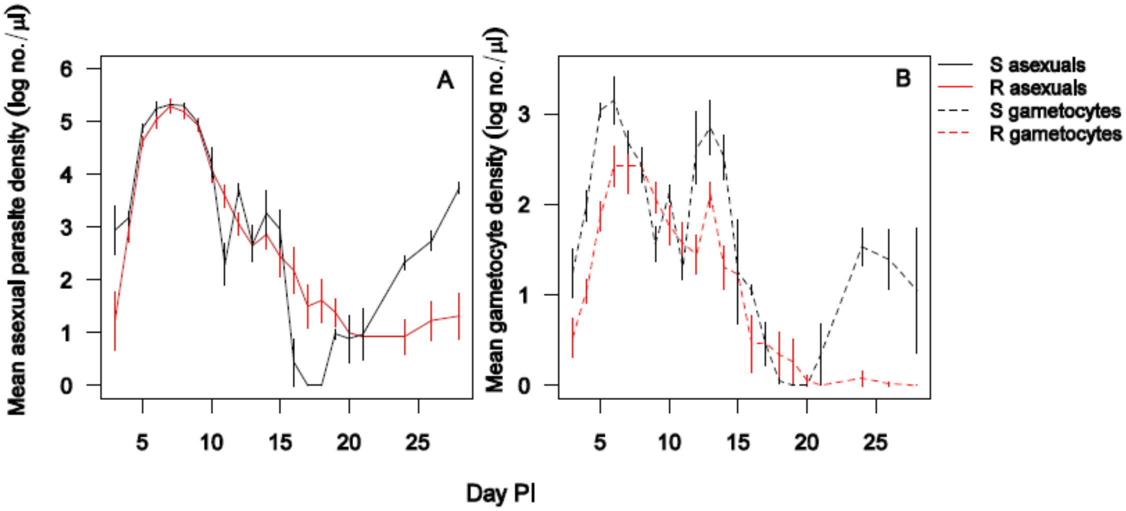

Figure S3. Asexual parasite (A, solid lines) and gametocyte (B, dashed lines) dynamics of drug-susceptible clone S (black lines) and drug-resistant clone R (red lines) in untreated single-clone infections. Data are means (± standard error) of 6 mice (clone R) and 3 mice (clone S). Data from (Huijben *et al.*, 2010).

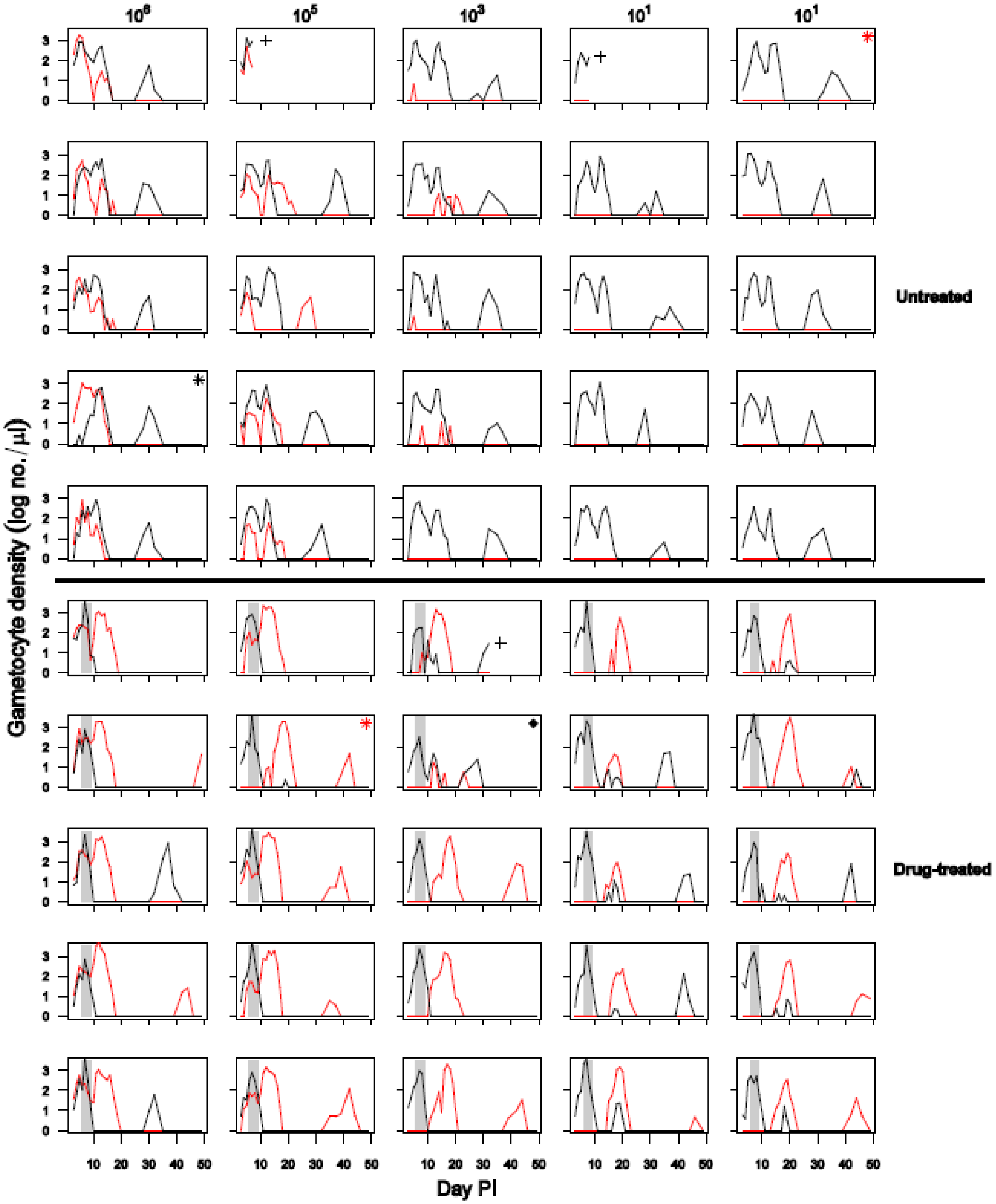

Figure S4. Gametocyte densities of drug-sensitive clone S (black lines) and drug-resistant clone R (red lines) in mixed infections that were untreated (upper half) and drug treated (lower half). Drug treatment was given on days 6-9 post-infection as indicated by the shaded area. Infections were inoculated with 10^6 parasites of clone S and 10^6 (left column), 10^5 (second column), 10^3 (middle column) or 10^1 (last two columns) parasites of clone R. Only mice included in the analysis are shown (see Table S1).

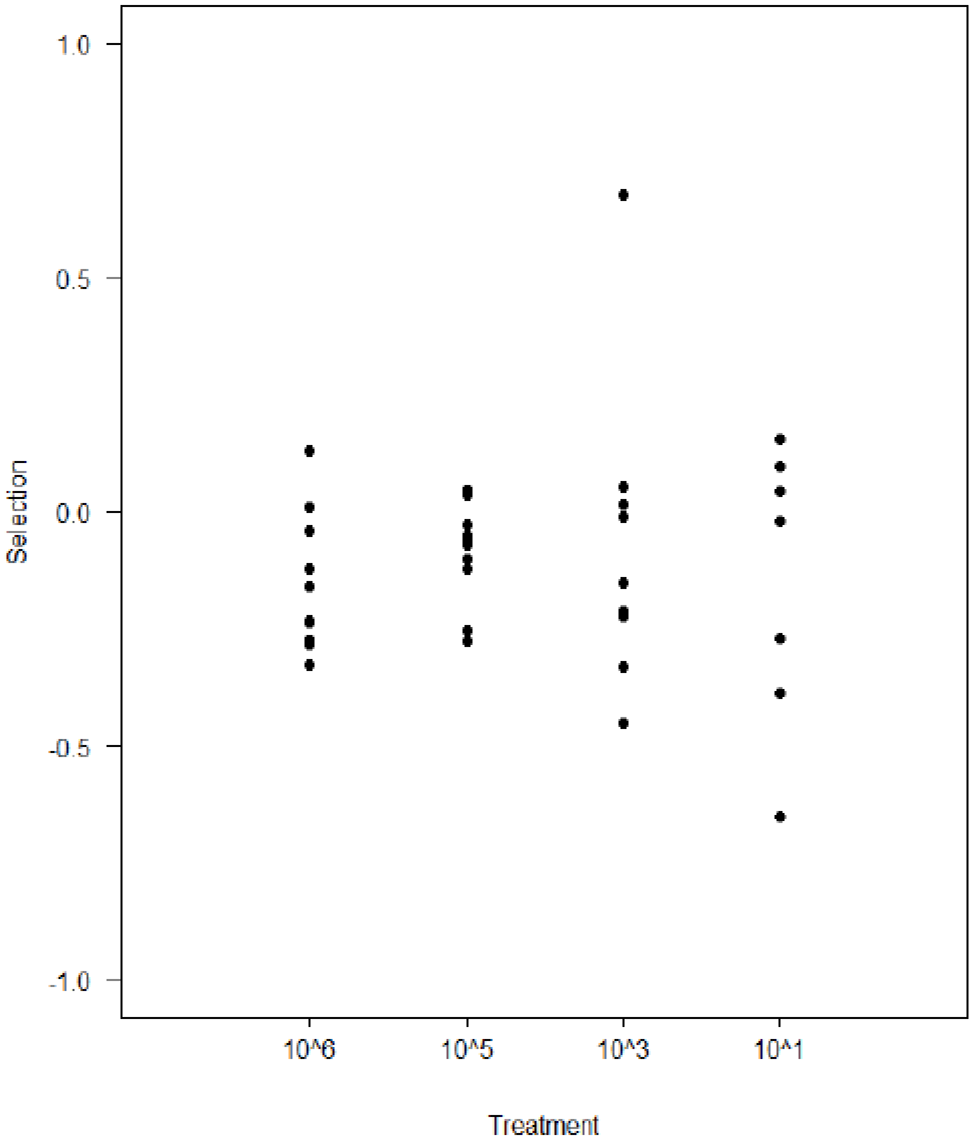

Figure S5. Selection coefficients of clone R during the log-linear growth phase (day 3-6 post-infection) for mixed infections with clone R inoculum of 10^6, 10^5, 10^3 and 10^1 parasites and clone S an incolulum size of 10^6 in all cases. Treated and untreated groups were pooled as growth rates were estimated on densities prior to treatment, leading to up to 10 mice in each group of 10^6, 10^5, 10^3 clone R inocula and up to 20 mice in the 10^1 inoculum group. Successful selection coefficient estimation was not possible for all mice, particularly in the 10^1 group, due to exclusion of mice (Table 1) and not having a minimum of two density measures of both clones between days 3 and 6.

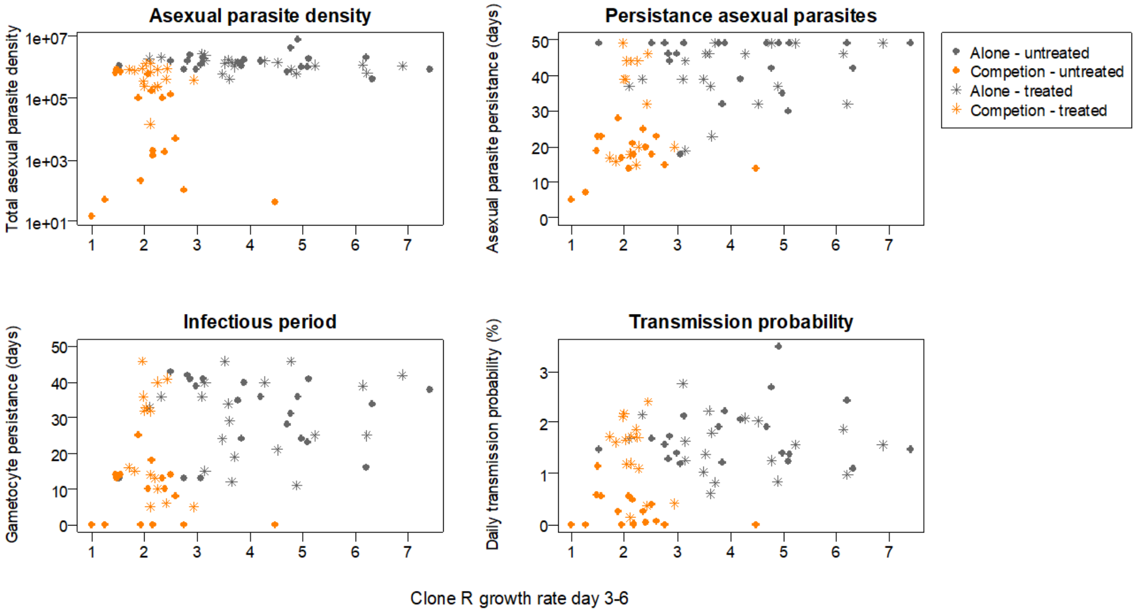

Figure S6. Association between initial growth rate of clone R (days 3-6 post-infection) with four clone R fitness parameters: Total asexual parasite density (top left), asexual parasite persistence (top right), infectious period (bottom left) and transmission probability (bottom right). Each symbol represents a single mouse, orange symbols represent mixed infections, and grey symbols are single R clone infections. Circles are untreated infections, asterisks are drug treated (days 6-9 post-infection) infections.

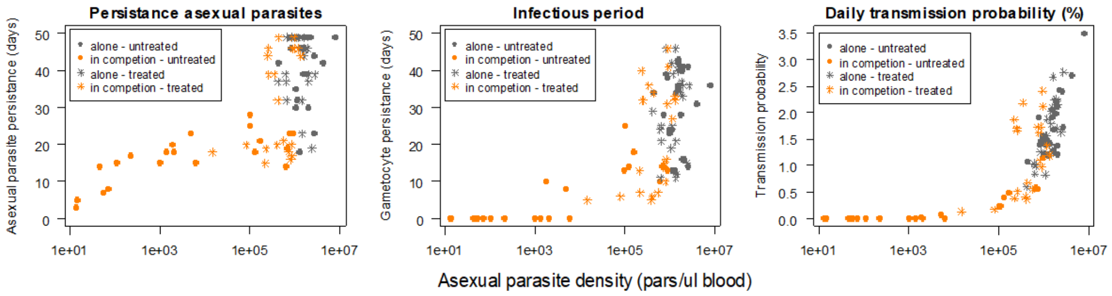

Figure S7. Association between total asexual parasite density of clone R with fitness parameters asexual parasite persistence (left), infectious period (middle) and daily transmission probability (right). Each symbol represents a single mouse, orange symbols represent mixed infections, and grey symbols are single R clone infections. Circles are untreated infections, asterisks are drug treated (days 6-9 post-infection) infections.

Table S1. A) Infection parameters of clone R in untreated infections when alone or with drug-sensitive parasites. Mean (±sem) summed asexual parasite density over whole infection period, mean asexual parasite persistence, mean infectious period and mean transmission probability of untreated infections either alone or in competition and the fold change of competition relative to single infection. B) Infection parameters of clone R in mixed infections. Mean (±sem) summed asexual parasite density over post-drug treatment period (from day 10 PI), mean asexual parasite persistence, mean infectious period and mean transmission probability of untreated infections over post-drug treatment period of untreated and drug treated infections and the fold change of treatment over non treatment. C) Mean (±sem) summed asexual parasite density and mean transmission probability in untreated and drug treated infections over whole infection period. D) Estimated growth rates (fold increase/day) of resistant parasites between days 3-6 post-infection in untreated and drug treated infections. E) Mean relative growth rates of clone R in competition (±sem). F) Statistical model output of strain-effect on inoculum-effect on growth rates between clone S and clone R .

(A) Untreated infections – whole infection period

	Asexual parasite density (daily no. parasites/μl blood)			Persistence asexual parasites (days)			Infectious period (days)			Transmission probability (%)		
	Alone	In comp	Fold change	Alone	In comp	Fold change	Alone	In comp	Fold change	Alone	In comp	Fold change
10^6	3.07 ± 1.2	0.72 ± 0.06	0.23	47.4±3.1	19.8±2.1	0.42	40.6±1.2	12.8±1.0	0.31	1.9±0.4	0.7±0.1	0.37
10^5	1.71 ± 0.61	0.12 ± 0.01	0.07	38.8±3.8	23.0±2.2	0.59	26.4±5.6	17.5±2.7	0.66	1.8±0.3	0.3±0.1	0.20
10^3	0.99 ± 0.24	0.0020 ± 0.008	0.002	41.6±5.5	18.6±1.5	0.45	28.0±3.9	3.6±2.2	0.13	1.4±0.1	0.03±0.01	0.019
10^1	1.62 ± 0.16	0.0010 ± 0.007	0.0006	41.5±1.0	10.6±1.9	0.26	26.8±3.3	0±0	1/∞	1.8±0.1	0±0	1/∞

(B) Mixed infections – post-treatment period

	Asexual parasite density (x 10^4 parasites/ μl blood)			Persistence asexual parasites (days)			Infectious period (days)			Transmission probability (%)		
	Untreated	Drug treated	Fold change	Untreated	Drug treated	Fold change	Untreated	Drug treated	Fold change	Untreated	Drug treated	Fold change
10^6	0.15±0.02	5.9±0.8	40	10.8±2.1	20.2±7.5	1.9	5.8±0.9	19.4±7.0	3.4	0.1±0.007	1.6±0.2	14
10^5	0.36±0.08	12.9±3.0	36	14.0±2.2	25.3±6.5	1.8	11.0±2.5	24.5±5.7	2.2	0.2±0.05	2.0±0.1	8.2
10^3	0.07±0.06	115±16.6	1651	9.6±1.5	27.0±8.0	2.8	3.2±2.0	25.0±7.5	7.8	0.02±0.02	1.4±0.04	55
10^1	0.09±0.08	43.9±11.1	490	3.3±1.3	22.7±4.3	14.0	0±0	16.0±4.1	∞	0±0	0.7±0.1	∞

(C)Treated mixed infections – whole treatment period

	Asexual parasite density (x 10^6 parasites/ µl blood)			Persistence asexual parasites (days)			Infectious period (days)			Transmission probability (%)		
	Untreated	Drug treated	Fold change	Untreated	Drug treated	Fold change	Untreated	Drug treated	Fold change	Untreated	Drug treated	Fold change
10^6	0.72 ± 0.06	0.84 ± 0.04	1.2	19.8±2.1	29.2±7.5	1.5	12.8±1.0	26.4±7.0	2.1	0.7±0.1	1.9±0.1	2.7
10^5	0.12 ± 0.01	0.26 ± 0.03	2.1	23.0±2.2	34.3±6.5	1.5	17.5±2.7	30.3±6.0	1.7	0.3±0.1	1.9±0.1	5.3
10^3	0.0020 ± 0.008	1.15 ± 0.17	562	18.6±1.5	36.0±8.0	1.9	3.6±2.2	25.0±7.5	6.9	0.03±0.01	1.2±0.03	43
10^1	0.0010 ± 0.007	0.44 ± 0.11	462	10.6±1.9	31.7±4.3	3.0	0±0	16.0±4.1	∞	0±0	0.6±0.1	∞

(D) Growth rates clone R alone and in competition

	Alone	In competition	Fold change
10^6	3.20 ± 0.32	1.85 ± 0.11	0.58
10^5	3.77 ± 0.23	2.17 ± 0.07	0.58
10^3	5.72 ± 0.39	2.53 ± 0.29	0.44
10^1	4.00 ± 0.36	2.06 ± 0.27	0.52

Stats: Competition: $F_{1,64}$=92***, R-inoculum: $F_{3,64}$=11***, Comp*R-inoc: $F_{3,64}$=3.9*

(E) Relative growth rates clone R in competition

	Untreated+treated (prior to treatment)
10^6	0.88±0.05
10^5	0.93±0.03
10^3	1.05±0.14
10^1	0.89±0.09

Stats: R-inoculum: $F_{3,28}$=0.87

(F) Growth rate strain effect:

Strain: $F_{1,56}$= 1.6, R-inoculum: $F_{3,56}$= 4.0*, Strain*R-inoc: $F_{3,56}$=0.4 (Excluding interaction: Strain: $F_{1,59}$= 1.6, R-inoculum: $F_{3,59}$= 4.0*)
